# Environment-Living Organism's Interactions from Physiology to Genomics

**DOI:** 10.1155/2015/270736

**Published:** 2015-10-07

**Authors:** Shao Hongbo, Chen Sixue, Marian Brestic

**Affiliations:** ^1^Jiangsu Key Laboratory for Bioresources of Saline Soils, Provincial Key Laboratory of Agrobiology, Institute of Agro-Biotechnology, Jiangsu Academy of Agricultural Sciences, Nanjing 210014, China; ^2^Key Laboratory of Coastal Biology & Bioresources Utilization, Yantai Institute of Coastal Zone Research (YIC), Chinese Academy of Sciences (CAS), Yantai 264003, China; ^3^Cancer and Genetics Research Complex, University of Florida, 2033 Mowry Road, Gainesville, FL 32610, USA; ^4^Department of Plant Physiology, Slovak Agricultural University, Tr. A. Hlinku 2, 949 76 Nitra, Slovakia

Human beings depend on the environment for stable survival and sustainable development, so the environment and organisms should be one bi-interacting whole body. Environment-living organism's interactions in terms of physiology, molecular biology, and genomics are the essential base for establishing and crossing different disciplines where life sciences link with environmental sciences fully and they have resulted from many important subjects such as soil biology, ecophysiology, molecular ecology, and environmental genomics. These disciplines support current human beings' sustainable development by providing new ways to cope with nature.

With global economic change, large-scale urbanization, and environmental pollution, human beings face more serious challenges. To solve these problems and search for new ways we must understand environment-living organism's interacting responses by these disciplines, to which less attention has been paid for the past 20 years. In this special issue for this journal we have solicited papers from experts working with various aspects of genomic research in relation to plant biology, soil biology, agricultural sciences, aquaculture, and environmental protection. We invited researchers to present their novel results that can deeply understand environment-organisms' interactions. The special issue is dedicated to the crossing disciplines among plant biology, soil biology, microbiology, agricultural sciences, aquaculture, and ecorestoration by covering the following main aspects: (i) plants from physiology, molecular biology, and metabolisms to genomics; (ii) plant-soil interactions in terms of molecular responses; (iii) organisms-based ecorestoration; (iv) molecular microbiology related to agricultural sciences, aquaculture, and environmental protection.

In the special issue, N. Yi et al. established the linkage of water properties with the abundance and diversity of* Eichhornia crassipes* by comparative genomics and precise environmental biology methodology, providing the clear evidence for* Eichhornia crassipes* to remove nitrogen in different effluents around Dianchi Lake, China. These results can be used extensively for phytoremediation. T. Wang et al. firstly reported the results about efforts of copper nanoparticles on juvenile* Epinephelus coioides in terms of* growth parameters, digestive enzymes, body composition, and histology, providing more valuable parameters as environmental biomarkers in aquaculture and its management. G. H. Yu et al. used* SbPIP1*-transformed wheat seedlings as the materials to compare the related salt resistance. The result indicated that* SbPIP1* plays an important role in the salt stress response. Overexpression of* SbPIP1* could be used to improve the salt tolerance of important crop plants. The abiotic stresses like salinity, drought, and low and high temperature negatively affect plant growth and productivity. Dehydration-responsive element binding (DREB) transcription factor (TF) plays a key role for abiotic stress tolerance in plants. X. Zhang et al. reported the cloning and characterization of the SsDREB cDNA from* Suaeda salsa*. Its expression pattern was investigated in response to exogenous ABA, salt, cold, and drought stress treatments. Overexpression of this cDNA in transgenic tobacco led to enhanced tolerance to salinity and dehydration stresses. These integrated data suggest that the* SsDREB* transcription factor is involved in the regulation of salt stress tolerance in tobacco by the activation of different downstream gene expression. Unlike animals, plants are not mobile organism and cannot go away from adverse environmental conditions. Owing to these reasons, they create special system to adjust themselves in external stress conditions through instant transmit signals. Due to the temporary fluctuations in cytosolic calcium concentration, plant cells receive the signals from external stimuli, so they can accept the signals using their own machineries and decode the signal to secondary messenger. Calcium is broadly well known as a ubiquitous secondary messenger because of its diverse functions in plants. Ca^2+^ is encoded in various stimuli of abiotic and biotic stresses. Abiotic stresses caused by high magnesium, high sodium, low potassium, low phosphorous, ABA, and others affect the rate of germination, photosynthesis, seedling growth, leaf expansion, total biomass accumulation, and overall growth effects of plants. In recent decades, calcineurin B-like protein- (CBL-) interacting protein kinase (CIPK) complex is widely accepted as Ca^2+^ signaling mechanism, which is involved in response to different external stresses signals (Shao et al. 2008). In adverse stresses conditions, plants evolve a stress signal that is specifying Ca^2+^ signature. The specific Ca^2+^ signatures are received by closely controlled activities of plasma membrane and other organelles channels and transporters. In addition, this signature binds to EF hands domains of the CBL proteins. Consequently, the CBL proteins bind the NAF/FISL domain of C-terminal of the CIPK thus stimulating the kinase. On the other hand, N-terminal of the CBL protein directs the CBL-CIPK system to an exact cellular target region ensuing in the stimulated CIPK phosphorylating the proper target proteins. S. M. N. Manik et al.'s review recapitulated the recent and ongoing progress of positive ions (Mg^2+^, Na^+^, and K^+^), negative ions (NO_3_^−^, PO_4_^−^), and hormonal signaling, which are evolving from accumulating results of analyses of CBL and CIPK loss or gain of functions experiments in different species. Generally, this review provided further insights into the calcium sensor CBL-CIPK. Other important progresses can also be witnessed in the special issue.

All the results from the accepted papers have greatly contributed to the special issue, speeding up the extensive investigation of environment-living organism interactions. With the comprehensive application of modern methodology, more attention will be paid to this interesting and important field and it will further witness new advances.

